# Congenital transpositions of stomach and spleens with partial pyloric stenosis

**DOI:** 10.1259/bjrcr.20150196

**Published:** 2016-11-03

**Authors:** Benard Ohene Botwe, Benjamin Dabo Sarkodie, Yaw Boateng Mensah, William Kwadwo Antwi

**Affiliations:** ^1^Department of Radiography, School of Biomedical and Allied Health Sciences, University of Ghana, Accra, Ghana; ^2^Department of Radiology, University of Ghana School of Medicine and Denstistry, Accra, Ghana; ^3^Department of Radiology, Korle bu Teaching Hospital, Accra, Ghana

## Abstract

The occurrence of chromosomal aberrations resulting in congenital transposition of internal organs is rare. Isolated congenital stomach and spleen (multiple) transposition with partial pyloric stenosis, where the rest of the internal organs remain in their normal positions, to the best of our knowledge has not been reported before. Attention to, knowledge and records of this case should be considered vital for understanding future symptoms and occurrences and also for prevention of surgical mistakes.

Congenital transposition of internal organ(s) through the sagittal plane is rare, and may occur as a result of abnormal chromosomal aberrations.^1^ Complete transposition of the viscera, or situs inversus totalis, in which the thoracic and abdominal viscera occupy a position, the reverse of normal, occurs in 1 out of 10,000 live individuals.^1^ Situs inversus partialis involving single organs of which dextrocardia is most common, occurs less frequently than situs inversus totalis.

It has been reported that 25% of patients with situs inversus have complications of Kartagener syndrome which is a subgroup of primary ciliary dyskinesia.^2^ A variety of gastrointestinal abnormalities are also associated with individuals with situs inversus. These anomalies may involve the liver, biliary tract, stomach, spleen and the intestines.^3^ However in most cases, the transposition does not result in functional problems and patients live normal lives.^1,2^

Situs inversus of the stomach alone, also referred to as dextrogastria, is considered the rarest single organ transposition of all viscera.^4^ It has an incidence rate of less than 1:100,000.^4^ Hypertrophic pyloric stenosis also has an incidence of about 2–4 per 1000 live births.^5^ To the best of our knowledge, the combination of situs inversus of the stomach and multiple spleens combined with partial pyloric stenosis as reported herein has not been published before.

## Case report

**Figure 1.**
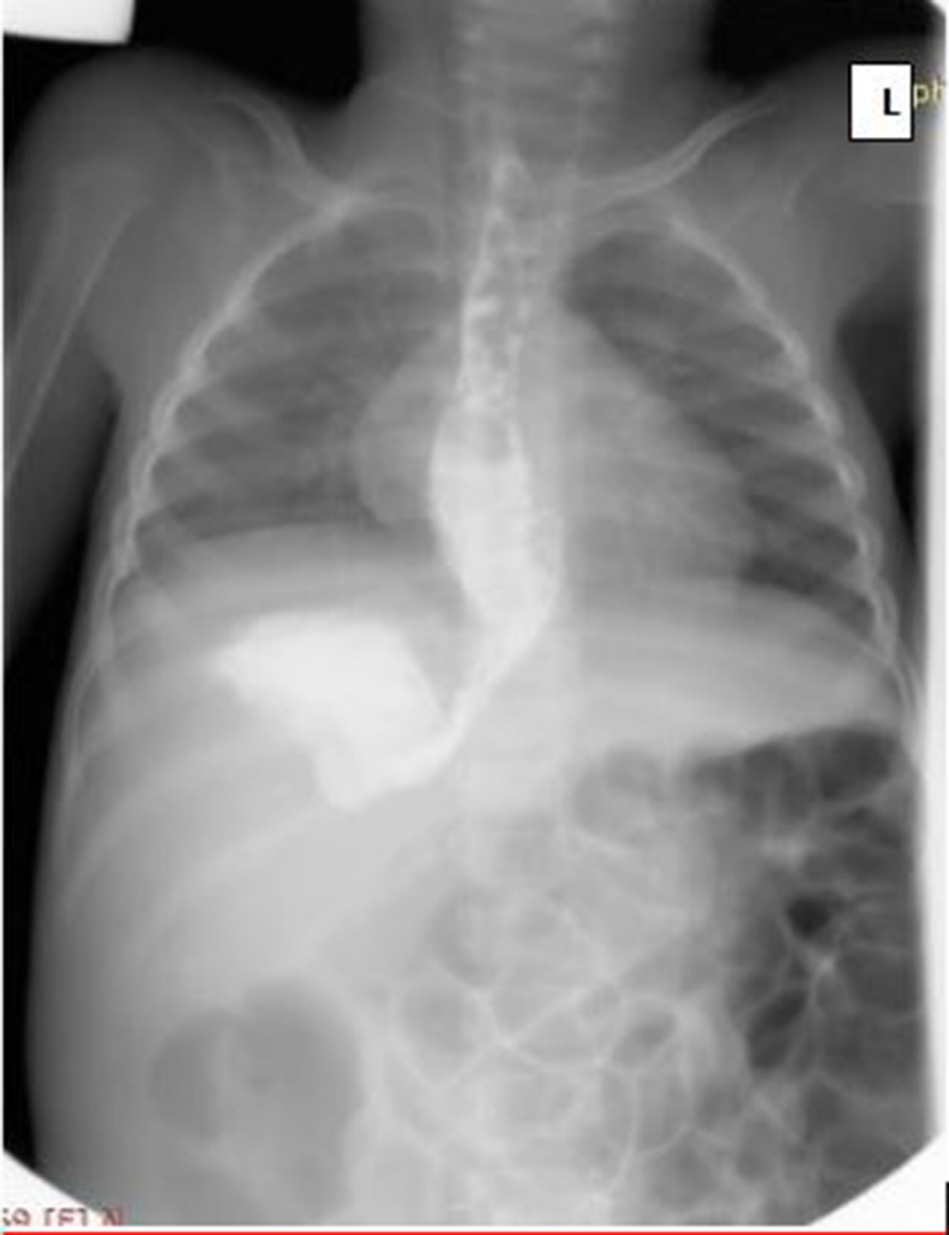
Demonstrating stomach on the right.

A 2 year old male child with a history of recurrent projectile non-bilious vomiting, since 4 weeks after birth, was referred for a barium meal study to rule out pyloric stenosis or gastro-oesophageal reflux disease. The patient experienced persistent hunger and was always underweight. The results of a full blood count test were within normal limits. A barium examination localised the stomach in the right upper quadrant of the abdomen, on the same side as the liver (Figure 1).

**Figure 2.**
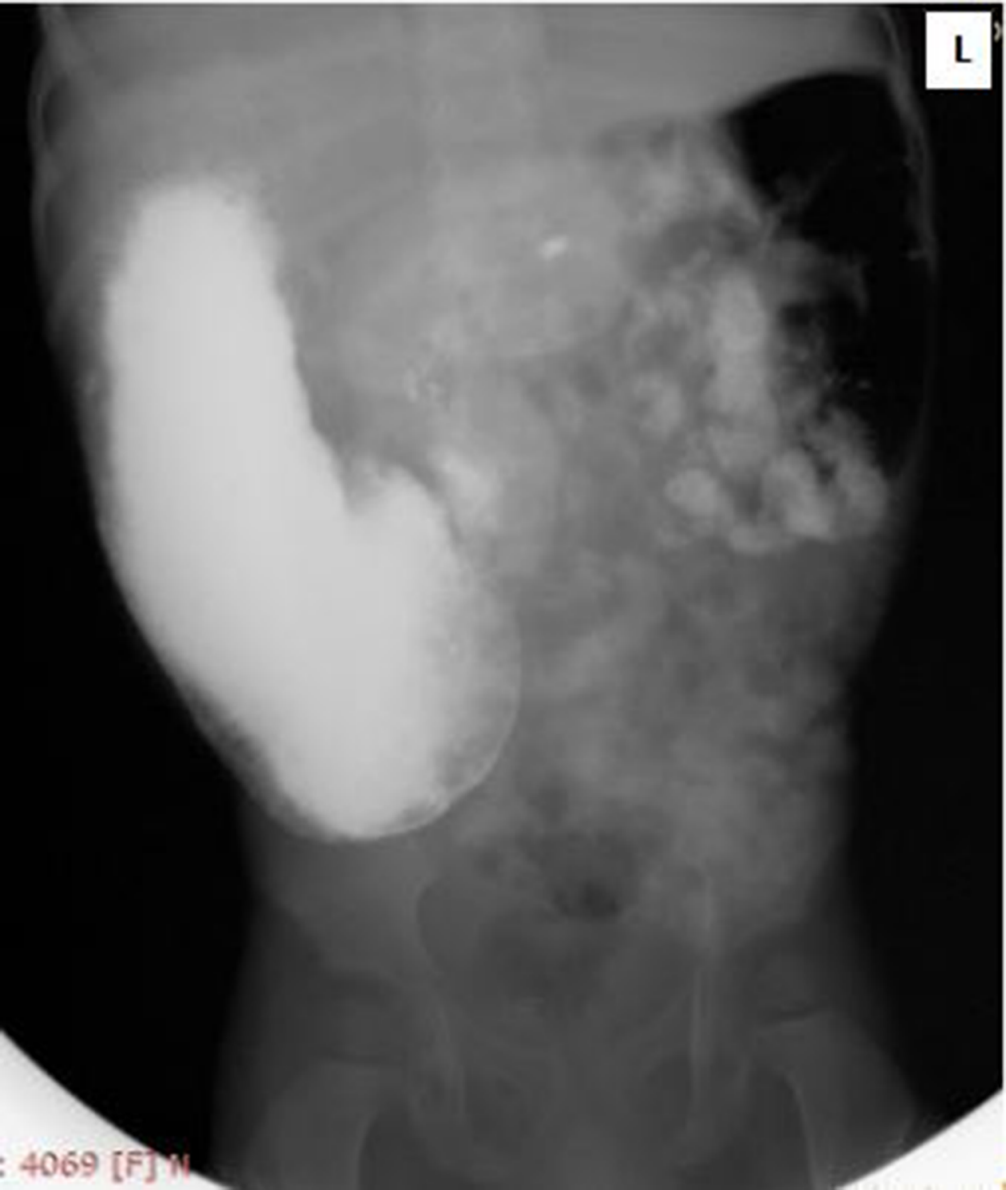
A 2-h barium meal radiograph showing distended stomach with partial pyloric stenosis.

Subsequent images revealed a distended stomach with indented gastric antrum, narrowed pylorus and delayed emptying suggestive of partial pyloric stenosis (Figure 2).

**Figure 3.**
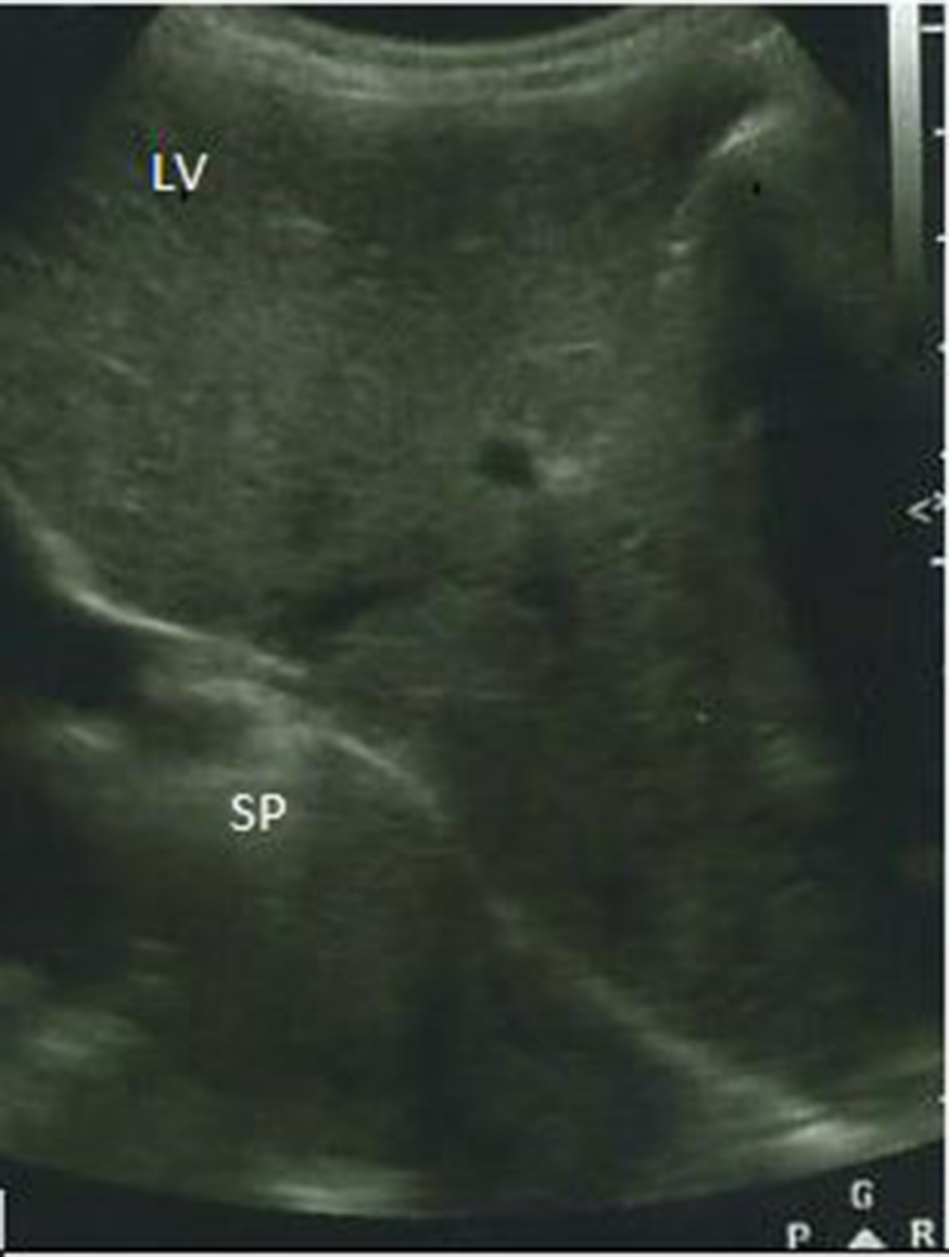
Ultrasound scan showing the SP adjacent to the LV. SP, spleen; LV, liver.

Complementary ultrasound examination localised the spleen (which showed no abnormality) and two other structures with the same appearance as the spleen in the right upper quadrant adjacent to the right kidney (Figure 3). The suspicion of other abnormalities and congenital transpositions of other organs lead to suggestions for other radiological procedures. Although MRI scan is the standard reference for definitive diagnosis of organ transposition,^2^ the procedure was, however, not performed owing to breakdown of the MRI equipment at that time. Hence a CT abdominal scan was undertaken with parental consent.

**Figure 4.**
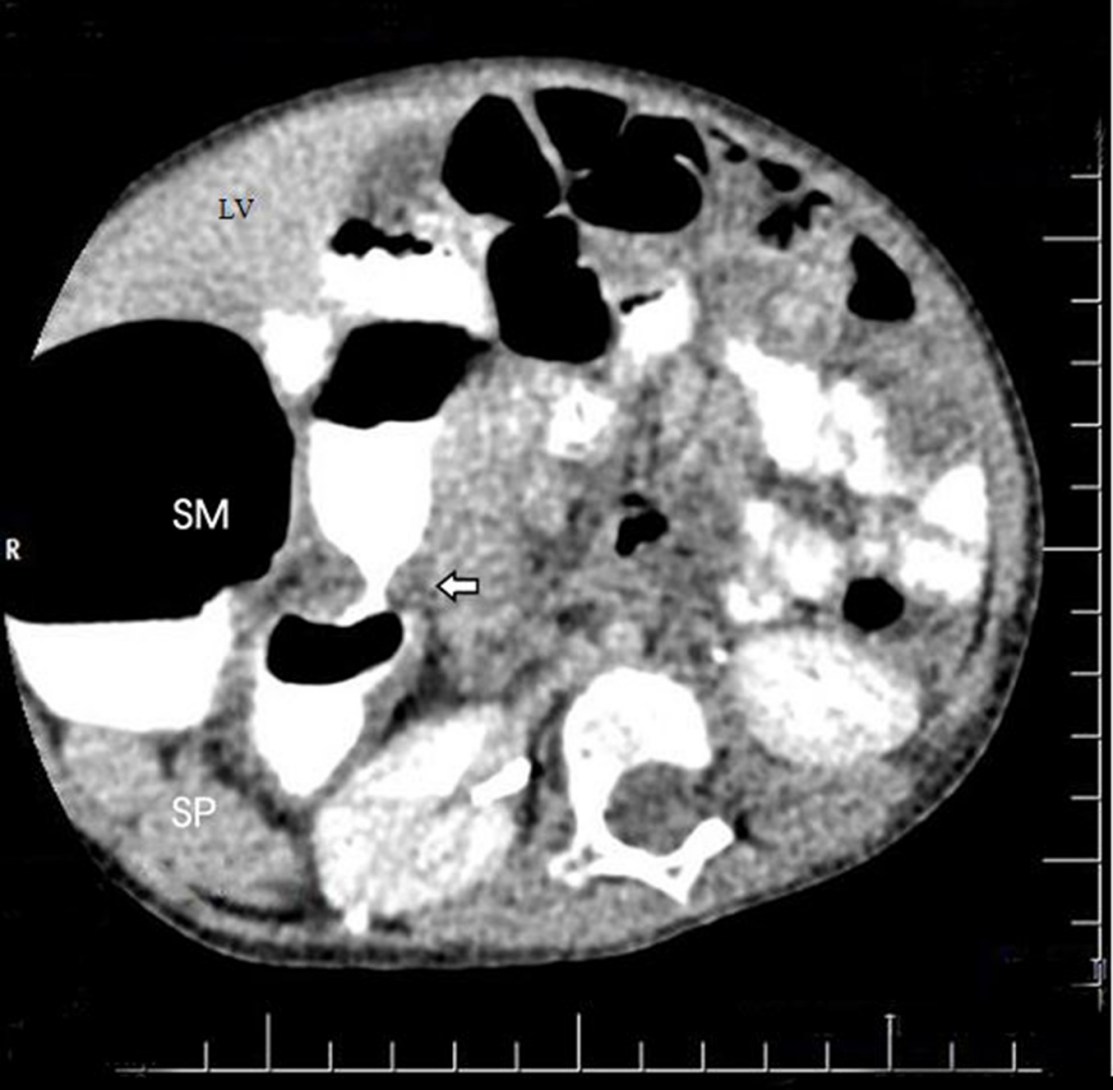
Contrast phase CT abdomen scan showing SM, LV and SP on the right. Arrow shows partial pyloric stenosis. SM, stomach; SP, spleen; LV, liver.

The CT scan confirmed the normal position of the internal viscera of the thorax and the abdomen, except the stomach and the spleen. The CT scan showed a dilated stomach on the right posterior to the liver and partial pyloric stenosis. The pyloric canal appeared elongated, and the whole pylorus was thickened. In addition, the spleen and the other structures with similar appearance as the spleen (suggesting multiple spleens) were found at the right side of the patient adjacent to the right kidney (Figure 4). A surgical correction of the partial pyloric stenosis further confirmed the anomaly.

**Figure 5.**
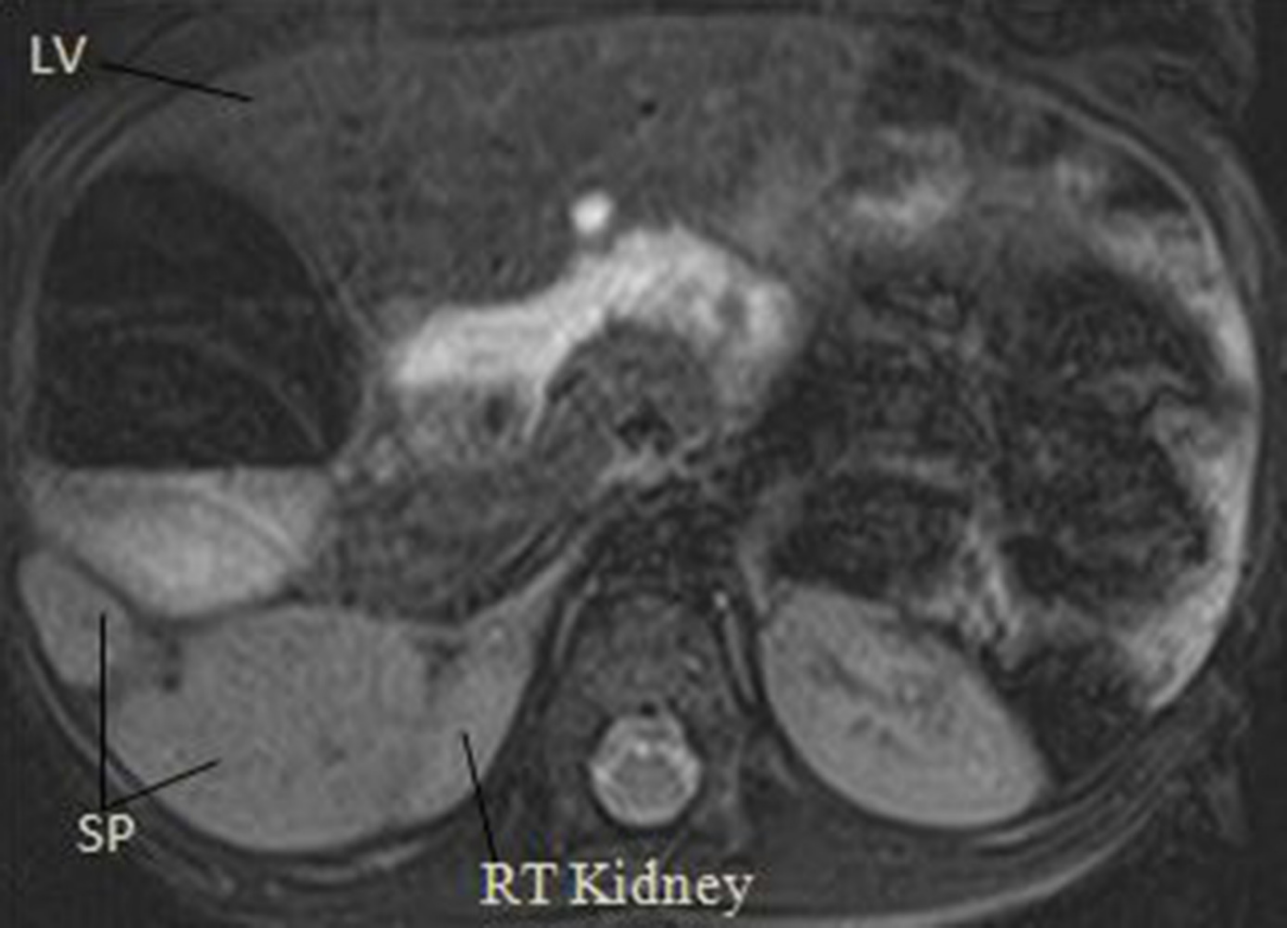
An axial free-breathing abdominal MRI scan showing multiple SPs and the RT. LV, liver; RT, right kidney; SP, spleen.

**Figure 6.**
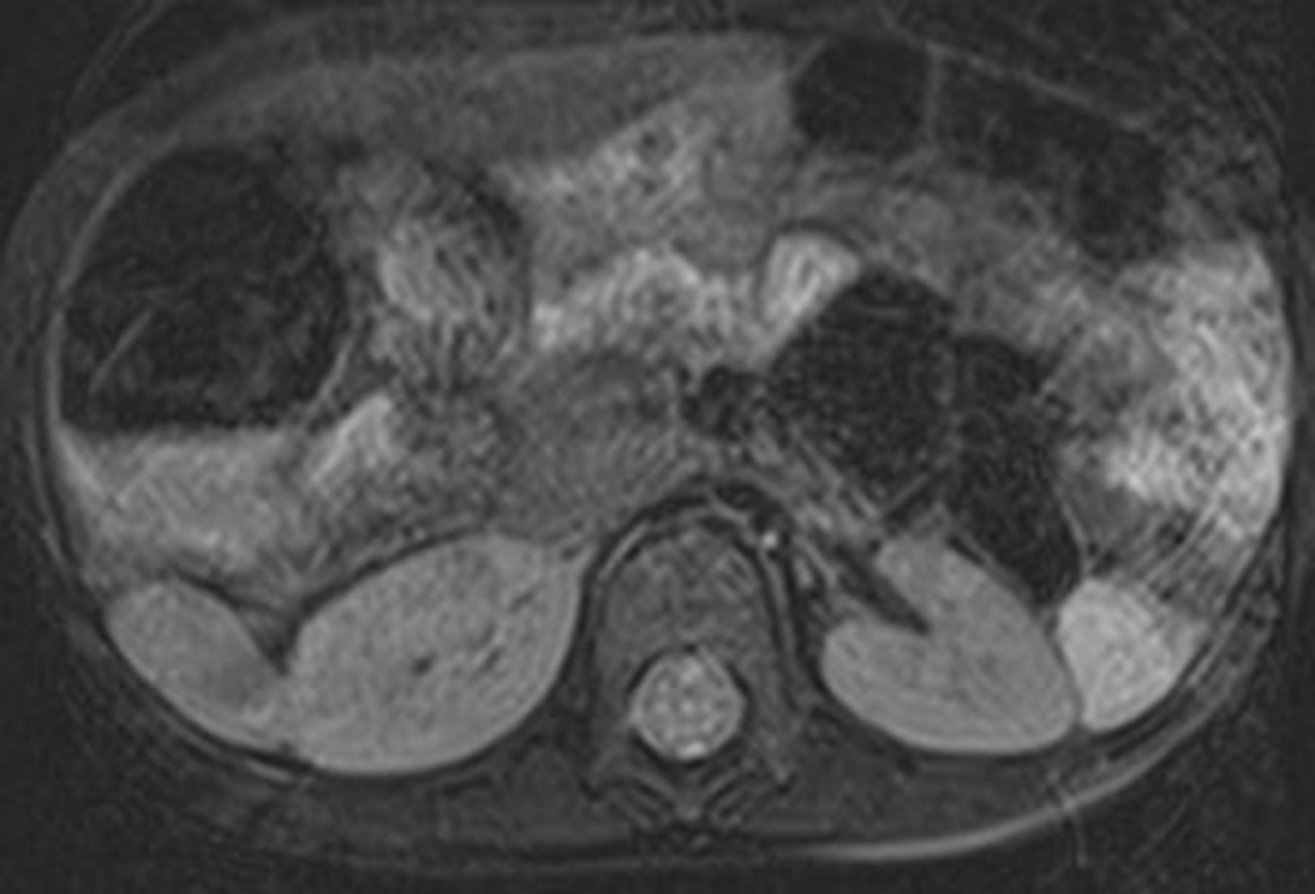
A free breathing MRI Axial view showing further the relationship between the right kidney and a spleen.

**Figure 7.**
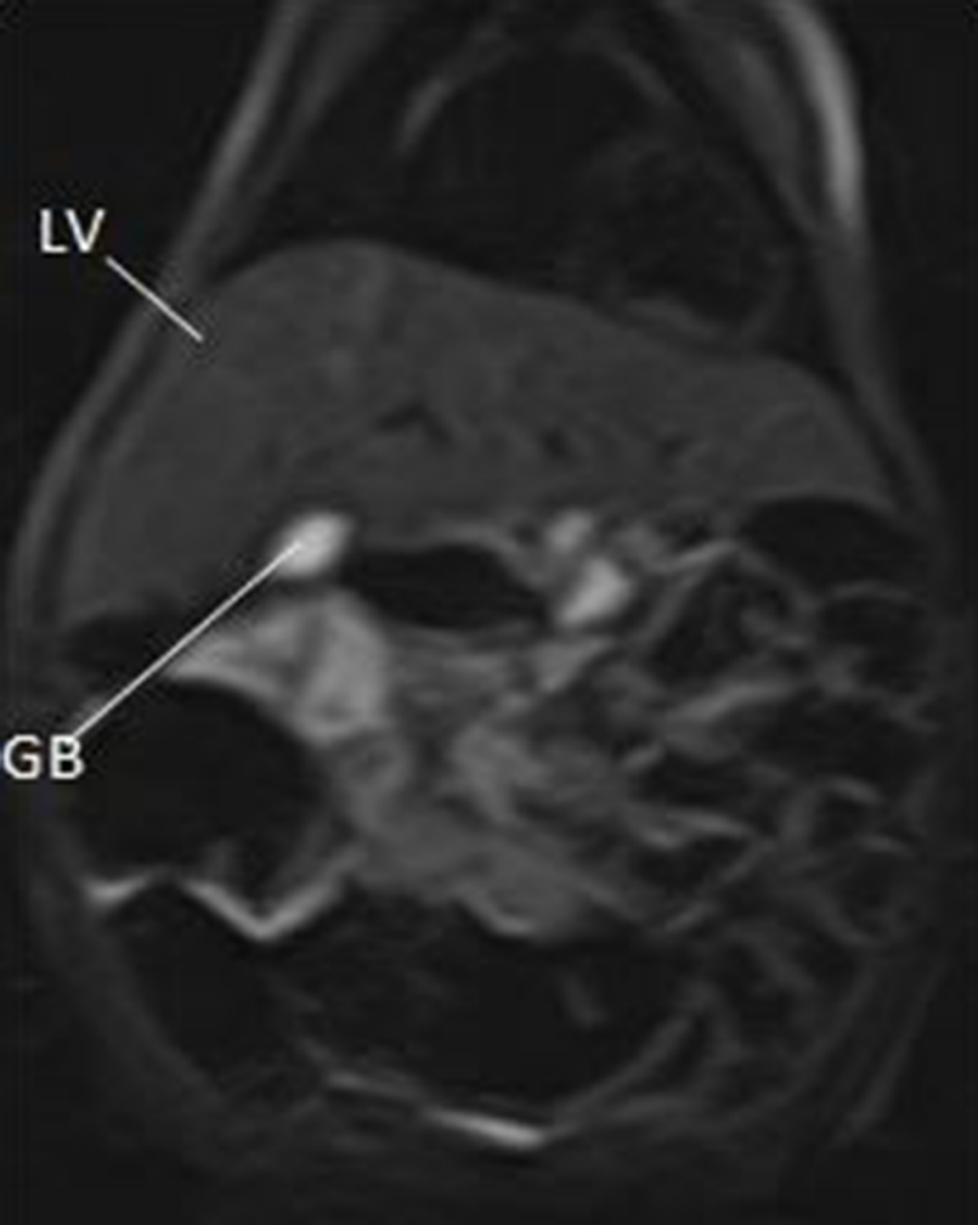
Showing the normal position of the LV and the GB. GB, gall bladder; LV, liver.

**Figure 8.**
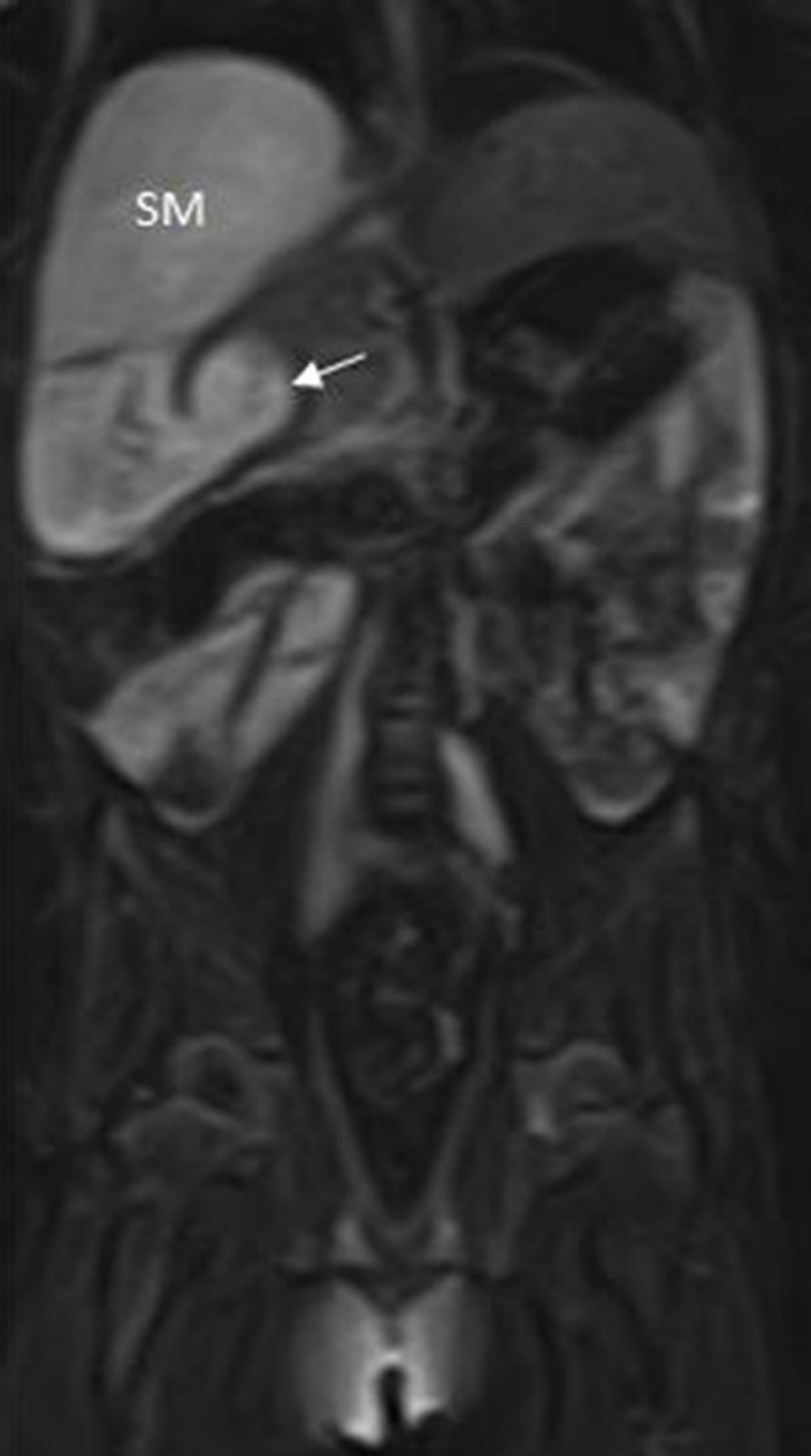
*T*_2_ coronal MRI view showing fluid-filled SM with thicken pyloric wall (arrow). SM, stomach.

**Figure 9.**
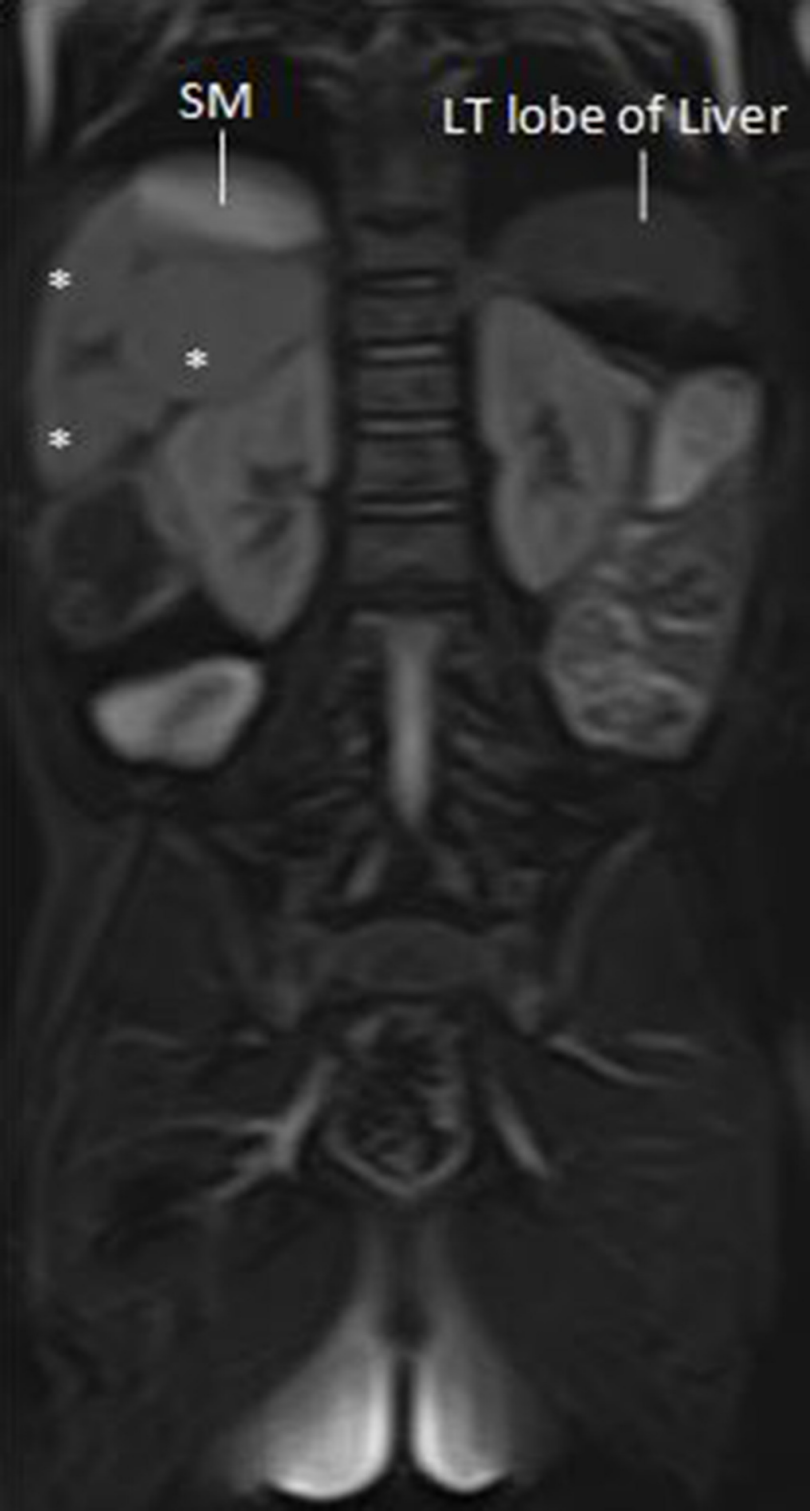
A *T*_2_ coronal MRI view showing multiple spleens (*) adjacent to the SM. SM, stomach; LT, Left.

A post-operative MRI scan performed 5 months later confirmed the anomaly as described above (Figures 5–9). The procedure was a free-breathing MRI scan as parental consent was without sedation. It also showed a thickened pyloric wall at the site of the repair and revealed the patient had multiple spleens (3) on the right (Figures 8 and 9). The child has had a normal life since the correction of the partial pyloric stenosis 2 years ago.

## Discussion

An extensive literature search showed no record of isolated cases of concurrent congenital transposition of the stomach and multiple spleens with partial pyloric stenosis. Though there are several aetiological theories about the condition, none has provided full explanation. Nonetheless, the condition has been suggested to result from chromosomal and embroyonic developmental aberrations. According to Supriya et al,^6^ the abdominal organs (mainly the intestinal tract, which develops from the midgut), are predominantly involved in malposition. However, the foregut and hindgut are considered to be more stable and fixed in their positions. The error in locating the foregut situated within the abdomen, that is, the stomach and duodenum down to the biliary papilla is excessively rare.^6^ The transposition of the abdominal viscera in the present case may be related to the malrotation of the gastrointestinal system during the embryonic development.^3^ In particular, the gastric tube failed to undergo its physiological rotation during the fourth week of the embryologic stage to situate the stomach and other organs in the left side of the abdomen.^7^

Other studies have also described the positive role of gut rotation determining factor and homeobox gene paired Like Homeodomain 2 (*Pitx2*) in the looping mechanism of the heart and the gut in such conditions.^8,9^ A report has also found that the parents of a situs inversus patient to be cousins, and this has supported the suggestion of chromosomal aberrations as an aetiological factor in situs inversus.^10^

Almy et al^11^ reported a case of inversion of the stomach alone. The right-sided stomach behind the left lobe of the liver was discovered during a cholecystectomy and later confirmed radiographically as situs inversus of the stomach. The majority of cases of right-sided stomach that have been described are also associated with eventration of the diaphragm owing to compression and congenital deformities of the diaphragm.^11,12^

Nawaz et al^12^ reported two extremely rare cases in which the situs inversus abdominus was associated with a congenital partial duodenal obstruction secondary to the duodenal diaphragm with a central aperture in one case, whereas the other child had a complete duodenal atresia as well as Fallot’s tetralogy. Similar to the case reported by Nawaz et al,^12^ the present case had dextrogastria with a congenital partial pyloric stenosis except that the duodenal diaphragm was normal. The liver, the gall bladder, the kidneys, the intestines and the heart in this case were normal and in their usual positions. The stomach was posteriorly related to the right lobe of the liver with the ligament of treitz. This position of the stomach was quite different from that observed in the case of Almy et al.^11^ There were also multiple spleens, which suggested a condition of polysplenia or Chaudhrey’s disease.

Several radiological modalities had to be used before arriving at the diagnosis for this child owing to the history provided by the referring doctor. However, ultrasound scan is useful as a first imaging modality while MRI scan is the standard reference for definitive diagnosis of organ transposition.^2^ It is known that most situs inversus are asymptomatic. In this case, the patient presented mainly with persistent vomiting for which a barium meal study was requested. The findings of this study is particularly very important for health professionals especially surgeons and emergency doctors to be aware of such conditions as modifications of surgical and interventional techniques to suit the mirrored image anatomy are needed. In particular, procedural problems could arise in laparoscopic cases. To further prevent mistakes in diagnosis and/or surgical intervention proper labelling of images preferably with lead letters should be enforced always.

Also the close relationship of the right-sided stomach to the liver may produce interesting and confusing changes in the liver ultrasound scan. Therefore, radiologists, radiographers and sonographers need to keep such anomalies in mind when evaluating children with the above condition.

## Conclusions

Although the presented example of a patient with congenital transposition of stomach and multiple spleens with partial pyloric stenosis is extremely rare, it can pose diagnostic, surgical and other interventional challenges. Careful evaluation of patients may assist in confronting this phenomenon and avoid mistakes. In this case, an imaging modality such as CT scan was additionally used to assess the organ transposition owing to equipment challenges; however, MRI scan is the standard reference for diagnosing this condition while ultrasound scan is useful as first imaging modality. Patients with such anomalies also need to be tagged to avoid interventional mistakes in cases of emergencies. Proper labelling of images preferably with lead letters during imaging is also crucial to prevent mistakes in diagnosis and/or surgical intervention.

## Learning points

1. Concurrent congenital transposition of the stomach and multiple spleens with partial pyloric stenosis is extremely rare.
2. When the anomaly occurs, the close relationship of the right-sided stomach to the liver may produce interesting and confusing changes in the liver ultrasound scan and radiologists and sonographers need to be aware of this.
3. Proper labelling of patients during radiological examinations and interventions preferably with lead letters need to be emphasized. Digital labelling after image acquisition has to be avoided.

## Consent

Written informed consent for the case to be published (including images, case history and data) was obtained from the patient’s parents for publication of this case report, including accompanying images.
